# Decrease in alcohol use disorder hospitalizations in Brazil: what does it mean?

**DOI:** 10.47626/2237-6089-2022-0588

**Published:** 2024-08-14

**Authors:** Renato Luís Pessôa, Alexandre Kieslich da Silva, Luiza Silveira Lucas

**Affiliations:** Faculdade de Medicina Universidade do Vale do Taquari Lajeado RS Brazil Faculdade de Medicina, Universidade do Vale do Taquari (UNIVATES), Lajeado, RS, Brazil.

**Keywords:** Alcohol use disorder, binge drinking, epidemiology, mental health services, public health

## Abstract

**Objective:**

To analyze the trend of hospitalizations for alcohol use disorder (AUD) in Brazil, by region, and establish its relationship with mental health care facilities.

**Methods:**

Data were collected through the Brazilian Unified Health System’s (SIH/SUS) Hospital Information System (Sistema de Informação Hospitalar) and the National Register of Health Establishments of Brazil (Cadastro Nacional de Estabelecimentos de Saúde [CNES]). We used linear regression models to estimate the effect of SUS psychiatric beds and Center for Psychosocial Care (Centro de Atenção Psicossocial [CAPS]) numbers on AUD hospitalizations.

**Results:**

From 2015 to 2020, 298,735 hospitalizations for AUD were recorded. Most of the patients admitted for AUD were male (88.8%). Individuals aged 60 years and older accounted for 11.7% of our cohort. The highest concentration of hospitalizations occurred in the South region (40.1%). The rate of hospitalizations per hospital bed remained relatively constant. The number of CAPS has a negative effect on SUS psychiatric beds in Brazil (average effect -22.31 [95% confidence interval {95%CI} -26.92, -17.70]). Psychiatric beds have a positive effect on AUD hospitalizations in the country (average effect 1.82 [95%CI 0.91, 2.74]).

**Conclusion:**

Prioritization guidelines for other forms of care are associated with a decrease in hospitalizations for AUD, so we highlight the importance of adequate training of health care professionals for proper referral of these patients to hospital admission when necessary.

## Introduction

Alcohol related disorders are one of the most prevalent psychiatric disorders worldwide, mostly affecting men.^1^ According to the World Health Organization (WHO), in 2016, harmful alcohol use accounted for 5.3% of all deaths and 132.6 million disability-adjusted life years (DALYs), and approximately half of this total of DALYs were related to non-communicable and mental health disorders. In Brazil, approximately one in every 24 adults has an alcohol use disorder (AUD), a proportion three times higher than in European countries such as Italy and Spain.^2^

Individuals with AUDs are also likely to seek medical help for acute episodes of alcohol poisoning. This is a serious condition, with the potential to affect almost any organ, in which the adverse effects of alcohol causing neurological, respiratory, cardiovascular, and gastrointestinal disturbances are the most clinically relevant. Acute intoxication in chronic alcohol users can also exacerbate potentially reversible problems such as electrolyte disturbances, thiamine deficiency, infection, and dehydration, which can lead to sequelae and death if not promptly corrected.^3^

Brazil’s Unified Health System (SUS) is meant to provide universal access to health care, including care for mental health and AUD. In 2001, Brazil’s Law 10,216, which provides for the protection and rights of people with mental disorders, was passed, and the Centers for Psychosocial Care (Centro de Atenção Psicossocial [CAPS]) were established as part of the SUS to offer an alternative to psychiatric hospitalizations.^4 , 5^ These specialist outpatient mental health services target people with severe disorders that require intensive care, and also work in integration with other health care units, admitting users in an inter-sectoral model.^6^ Consequently, a progressive reduction of SUS psychiatric beds began at the same time.^7^

Therefore, it is possible that the changes to the country’s mental health guidelines may have had a relevant influence on hospitalization of patients with AUDs. To investigate this hypothesis, the present study had several objectives: (a) to determine the trends in SUS AUD hospitalization, numbers of psychiatric beds, and numbers of CAPS in Brazil’s regions from 2015 to 2020; and (b) to analyze the associations between these variables.

## Methods

Data regarding hospitalizations were collected through the SUS Hospital Information System (Sistema de Informação Hospitalar [SIH]), while data on health establishments were extracted from Brazil’s National Register of Health Establishments (Cadastro Nacional de Estabelecimentos de Saúde [CNES]).^8^ Only hospitalizations from January 2015 to December 2020, coded as ICD-10 F10, for treatment of mental and behavioral disorders due to alcohol use were included in the study. Data from December of each year were used to analyze psychiatric bed and CAPS numbers. The rate of AUD admissions per psychiatric bed was calculated using the number of admissions as the numerator and the number of psychiatric beds as the denominator.

To perform an inclusive analysis, we analyzed data from all CAPS services, since Alcohol and Drugs CAPS (CAPS AD) are only set up in cities/regions with at least 70,000 inhabitants. Therefore, if the analysis had been restricted to CAPS AD then less populous cities would have been excluded from the analysis.^9^

### Ethical considerations

In accordance with Brazilian National Health Council (Conselho Nacional de Saúde) resolution number 466 of December 2012, Research Ethics Committee approval was unnecessary since the database used is in the public domain, with free and unrestricted access, and provides no data that allow identification of individual patients.

### Statistical analysis

We used regression models to assess the associations between psychiatric beds numbers, CAPS numbers, and AUD hospitalizations. We performed two simple regressions, using AUD hospitalizations as the dependent variable for psychiatric beds, and psychiatric beds as the dependent variable for CAPS. The average effect of CAPS on SUS psychiatric beds and the average effect of SUS psychiatric beds on AUD hospitalizations were estimated. Statistical analysis was performed using Stata v. 14.2.

## Results

**Figure 1:**
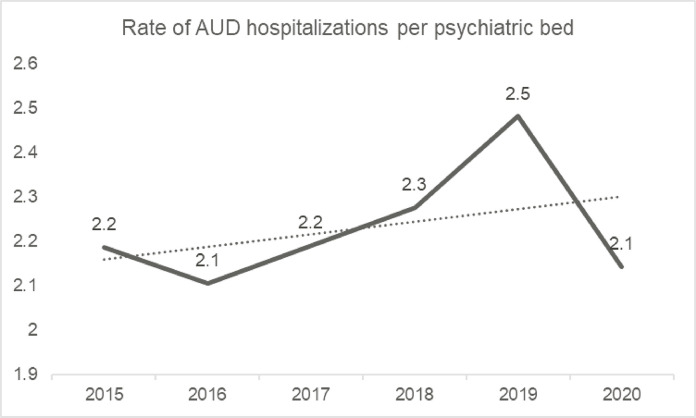
Rate of alcohol use disorder (AUD) hospitalizations per psychiatric bed, 2015-2020.

A total of 298,735 AUD hospitalizations occurring from January 1, 2015 to December 31, 2020, were analyzed in this study. Throughout the study period, male patients predominated (88.8%, n = 265,272 individuals) among the AUD hospitalizations reported in Brazil ( Table 1 ). Minorities of our cohort comprised women (11.2%) and individuals aged 60 years or older (11.7%). The decrease in the number of psychiatric beds in the SUS was progressive, with an overall rate of reduction from December 2015 to December 2020 of 33.4%. Over the same period, the number of CAPS increased by 14.9% ( Table 2 ). Additionally, the rate of AUD hospitalizations calculated per psychiatric bed was relatively stable ( Figure 1 ).

**Table 1:** Alcohol use disorder (AUD) hospitalizations by the Brazilian Unified Health System (SUS) in each of Brazil’s regions, with total number of admissions and minimum, maximum, average, and standard deviation per year, 2015-2020

Hospitalizations	Total	Minimum	Maximum	Average	Standard deviation
Total					
North	2,221	293	497	377.2	71.4
Northeast	50,424	7,443	9,525	8,477.7	635.9
Southeast	103,169	13,984	22,361	17,514.3	2,725.3
South	119,932	14,908	23,671	20,329.1	2,762.9
Midwest	22,989	3,182	4,986	3,905.4	630.4
					
Male sex					
North	1,956	257	442	326.0	67.9
Northeast	45,385	6,640	8,662	7,564.2	658.3
Southeast	89,451	12,144	19,619	14,908.5	2,569.8
South	108,825	13,424	21,537	18,137.5	2,910.1
Midwest	19,655	2,700	4,404	3,275.8	639.5
					
Female sex					
North	265	36	55	44.2	6.9
Northeast	5,039	738	1,011	839.8	101.0
Southeast	13,718	1,840	2,742	2,286.3	318.8
South	11,107	1,484	2,134	1,851.2	214.4
Midwest	3,334	482	633	555.7	52.4
					
Adults aged 60 and older					
North	226	23	46	37.7	8.4
Northeast	4,280	634	835	713.3	72.9
Southeast	13,680	1,926	2,738	2,280	273.6
South	14,377	1,809	2,612	2,396.2	303.4
Midwest	2,334	317	541	389.0	80.5

**Table 2:** Alcohol use disorder (AUD) hospitalizations, Brazilian Unified Health System (SUS) psychiatric beds, and Centers for Psychosocial Care (Centros de Atenção Psicossocial [CAPS]), 2015-2020

Region	AUD hospitalizations	SUS psychiatric beds (n)	CAPS (n)
North			
2015	497	390	175
2016	405	374	180
2017	374	358	187
2018	309	291	194
2019	293	326	198
2020	343	329	202
			
Northeast			
2015	9,525	5,668	947
2016	8,443	5,017	966
2017	8,101	4,523	1,008
2018	8,251	4,388	1,038
2019	8,661	3,940	1,041
2020	7,443	3,855	1,055
			
Southeast			
2015	22,361	14,777	974
2016	18,047	12,732	1,024
2017	15,716	11,359	1,071
2018	16,072	9,876	1,117
2019	16,989	8,610	1,148
2020	13,984	8,190	1,184
			
South			
2015	23,671	5,444	458
2016	21,830	5,411	452
2017	21,491	5,249	471
2018	19,920	5,221	479
2019	18,112	5,022	488
2020	14,908	5,004	491
			
Midwest			
2015	4,986	1,633	164
2016	4,110	1,536	165
2017	3,911	1,149	175
2018	3,397	1,292	185
2019	3,403	1,228	187
2020	3,182	1,221	191
			
Brazil			
2015	61,040	27,912	2,718
2016	52,835	25,097	2,787
2017	49,593	22,638	2,912
2018	47,949	21,068	3,013
2019	47,458	19,126	3,062
2020	39,860	18,599	3,123

Table 1 shows the results of the analysis of AUD hospitalizations, including total number of events for the period, average per year, and standard deviation. The South region had the highest incidence of total hospitalizations (40.1%) and accounted for the largest proportions of admissions of males (41%) and of individuals aged 60 years or older (41.2%). Hospitalizations of women were more prevalent in the Southeast region (41%). Despite its predominance in the number of hospitalizations, the South region had only 26.9% of the psychiatric beds and 15.7% of the CAPS in the December 2020 reports.

We used simple linear models to test the hypotheses of the effects of reducing psychiatric beds and increasing the number of CAPS on AUD hospitalizations. The result indicated that the number of CAPS has a negative effect on the number of SUS hospital psychiatric beds in Brazil (average effect -22.31 [95% confidence interval {95%CI} -26.92 to -17.70]) ( Table 3 ), and the number of SUS psychiatric beds has a positive effect on AUD hospitalizations in the country (average effect 1.82 [95%CI 0.91 to 2.74]) ( Table 4 ). This result indicates that AUD hospitalizations have a direct association with the number of SUS psychiatric beds, and an indirect association with CAPS.

**Table 3:** Average effect of CAPS on SUS psychiatric beds in Brazilian regions

	Adj. R^2^	Coefficient	95%CI	p-value
North	0.590	-2.82	-5.55 to -0.083	0.046*
Northeast	0.916	-15.05	-20.66 to -9.43	0.002^†^
Southeast	0.985	-32.04	-36.89 to -27.20	< 0.001^†^
South	0.924	-11.38	-15.41 to -7.36	0.001^†^
Midwest	0.537	-13.32	-27.50 to 0.861	0.060
Brazil	0.973	-22.31	-26.92 to -17.70	< 0.001^†^

95%CI = 95% confidence interval; CAPS = Centro de Atenção Psicossocial; Adj. R^2^ = adjusted R-squared; SUS = Brazilian Unified Health System.

* Reasonably strong evidence against the null hypothesis.

^†^ Very strong evidence against the null hypothesis.

**Table 4:** Average effect of SUS psychiatric beds on AUD hospitalizations in Brazilian regions

	Adj. R^2^	Coefficient	95%CI	p value
North	0.733	1.82	0.504 to 3.14	0.018*
Northeast	0.492	0.773	-0.114 to 1.66	0.073
Southeast	0.668	0.969	0.160 to 1.78	0.029*
South	0.825	15.52	6.83 to 24.22	0.008^†^
Midwest	0.568	2.76	-0.025 to 5.55	0.051
Brazil	0.857	1.82	0.913 to 2.74	0.005^†^

95%CI = 95% confidence interval; AUD = alcohol use disorder; Adj. R^2^ = adjusted R-squared; SUS = Brazilian Unified Health System.

* Reasonably strong evidence against the null hypothesis.

^†^ Very strong evidence against the null hypothesis.

## Discussion

After analyzing data from nearly 300,000 Brazilians admitted to hospital for AUD between 2015 and 2020, our results demonstrate that AUD hospitalizations decreased by 34.7% overall in Brazil. We also found an association between care indicators, specifically the number of SUS psychiatric beds and the number of CAPS, and the number of AUD hospitalizations. Higher numbers of CAPS are associated with lower numbers of SUS psychiatric beds, which are associated with fewer hospitalizations.

Although our study showed there was a decrease in the absolute number of AUD hospitalizations, in proportion to the number of psychiatric beds, there was relative stability. The decrease in hospitalizations may represent the establishment of prioritization guidelines for other forms of care, which recommend hospital admission only for cases in which the severity renders in-hospital treatment irreplaceable or when other measures have failed.^10^ In this context, access to community care alternatives, such as CAPS and therapeutic communities (TCs) run by the Ministry of Health was highlighted. Furthermore, as of 2012, receipt of funding for psychiatric beds is no longer dependent on the production record, which may favor under-recording of admission to these beds.^11^

Although these data emphasize a decrease in AUD hospitalizations, the real meaning of this information still needs to be clarified. Recent results published by other authors show an increase in alcohol abuse among adults in the main Brazilian capitals.^12^ Thus, we hypothesize that the decrease in AUD hospitalizations in association with the increase in the number of CAPS means that these patients are receiving treatment in social settings. This hypothesis is consistent with data indicating advances in the dehospitalization of psychiatric patients in Brazil.^13^

Another hypothesis is that these patients are placed in TCs, treatment units inside or outside the hospital environment recognized as part of the SUS mental health care network.^14^ Although there are regulations for the performance of TCs, guided by universal guidelines validated with scientific evidence, the existence of institutions that practice irregular activities and do not follow evidence-based approaches in Brazil points to the need for adequate supervision, regulation, and training of professionals with expertise in addiction disorders.^15^

In addition, TCs provide treatment for longer periods than the CAPS and standardized institutions are able to offer a longer abstinence period for patients, which is necessary for severe cases to learn skills to avoid relapse.^16^ The lack of temporal data regarding TCs in Brazil prevented their inclusion in our results.

Historically, there have been inequalities in the distribution of CAPS services among the regions of the country.^17^ Despite efforts to expand services, inequalities remain. The South region, responsible for the largest number of AUD hospitalizations had only 15% of the country’s CAPS in 2020. In the same period, the Northeast region had half the number of hospitalizations and 34% of the country’s services. Moreover, Brazil has about 324,000 municipalities with populations between 15 and 20 thousand inhabitants that do not have any CAPS services, showing that there is still a long way to go to universalize access to mental health in the SUS.^18^

Indeed, examining the average numbers of AUD hospitalizations reveals interesting differences among the Brazilian regions. Between 2015 and 2020, admissions for AUD patients were highest in the South (average > 18,000 per year) and Southeast (average > 14,000 per year) regions. Given that use of services is influenced by individual characteristics of the professionals/services, it is possible that factors regarding professional behavior, such as stigma and preparation to work in this area, have an influence on this inequality.^19^

In the 2013 National Health Survey, the South and Southeast regions had the lowest proportions of binge drinking among Brazilian regions (11.1 and 12.8% respectively) and two of the three highest proportions of regular drinking (28.4 and 24.1% respectively).^20^ In fact, the proportion of regular drinking was more compatible with hospitalization distribution among the regions in our study. Moreover, the Southern region has the highest prevalence of underage drinking, which is a predictor of risk for alcohol problems in adulthood.^21 , 22^ Thus, early onset drinking could be a determinant of the development of AUD in adulthood.

In the present study, hospitalizations were more frequent among male patients in Brazil. Worldwide, AUD is more frequent in men than in women.^23 - 25^ A systematic review linked this difference to socio-cultural sanctions, which affect women more than men.^25^ Thus, women may be less likely to seek treatment because of social stigma.^26^ Also, women feeling intoxicated with smaller amounts of alcohol could function as an inhibitory mechanism for excessive alcohol consumption.

According to the III National Survey on Drug Use by the Brazilian Population (III Levantamento Nacional sobre o Uso de Drogas pela População Brasileira) (2017), alcohol dependence was 3.4 times higher among men than women.^27^ However, the low number of hospitalizations among women in the period we studied (7.9 times lower than among men) highlights the social stigma among women with AUD, which may limit seeking help and/or access to social and treatment support networks. Consequently, there is an evident need to break this social barrier, since it is known that women develop the disorder in less time than men, and thus present more complications and more severe problems.^28^

Moreover, there were 34,897 (11.7%) hospitalizations of individuals aged 60 years or older in Brazil during the period studied. The prevalence of AUD in older people is lower than in younger people, but rates may be underestimated by under-identification or misdiagnosis. The presentation in this age group can be atypical, for example falls and confusion, and be confused with other comorbidities, which makes diagnosis difficult. However, severe disorders in older people who misuse alcohol are more common, and are associated with lower perceived health status and smaller social networks.^29^ Thus, more attention is needed at the primary, secondary, and tertiary levels of AUD prevention, considering the population growth of elderly individuals in society.

Developing AUD prevention strategies is critical because of its high prevalence and the elevated cost to the public health care system.^30^ Globally, programs have been developed for early screening of unhealthy alcohol use and optimal intervention.^31^ Mostly, the screening and care of the patient with AUD can be done in primary care, for which health professionals must have adequate training and support for these patients.^32^

### Limitations

Although this study reveals important data about AUD in Brazil, some limitations should be noted. The study is retrospective and the data used were secondary, extracted from official databases in Brazil. As already discussed, there is a possibility that cases are underreported. In addition, socio-behavioral factors may have an influence on AUD hospitalizations, which could not be assessed by our study. Considering our limitations, further research is needed to understand AUD hospitalizations, patterns and correlations.

## Conclusion

In conclusion, from 2015 to 2020, there was a decrease in hospitalizations for AUD in Brazil and the number of beds and CAPS had an overall effect. The highest numbers of hospitalizations were found in the South region and in males. Studies focusing on admission of these patients to other health services are necessary to estimate the real prevalence of the disease in the country. Finally, the study emphasizes the importance of training health professionals in proper referral of these patients to hospital admission when necessary.

## References

[1] Carvalho AF, Heilig M, Perez A, Probst C, Rehm J (2019). Alcohol use disorders. Lancet.

[2] World Health Organization (WHO) (2018). Global status report on alcohol and health 2018.

[3] Jung YC, Namkoong K (2014). Handb Clin Neurol.

[4] Brasil, Ministério da Saúde (2017). Portaria Nº 3.588. Diário Oficial da União.

[5] Nascimento Alves DS, Fagundes da Silva PR, Costa NR (2012). Advances and challenges of psychiatric reform in Brazil 22 years after the Caracas declaration. Medwave.

[6] Pinsky I, Bernal C, Vuolo L, Neighbors C (2018). Introducing care management to Brazil's alcohol and substance use disorder population. Braz J Psychiatry.

[7] Fernandes CJ, Ferreira De Lima A, Santos De Oliveira PR, Silva W, Santos D (2020). Índice de Cobertura Assistencial da Rede de Atenção Psicossocial (iRAPS) como ferramenta de análise crítica da reforma psiquiátrica brasileira. Cad Saude Publica.

[8] Brasil, Ministério da Saúde Tabnet - DATASUS.

[9] Brasil, Ministério da Saúde (2017). Portaria de consolidação Nº 3. Diário Oficial da União.

[10] Barros Teixeira M, de Leão Ramôa M, Engstrom E, Mendes Ribeiro J (2017). Tensions between approach paradigms in public policies on drugs: an analysis of Brazilian legislation in 2000-2016. Cien Saude Colet.

[11] Opaleye ES, Noto AR, Locatelli DP, Amato TC, Bedendo A (2021). II Relatório brasileiro sobre drogas: sumário executivo.

[12] Malta DC, da Silva AG, Prates EJS, Alve FTA, Cristo EB, Machado ÍE (2021). Convergence in alcohol abuse in Brazilian capitals between genders, 2006 to 2019: what population surveys show. Rev Bras Epidemiol.

[13] Guerrero AVP, Bessoni EA, Cardoso AJC, Vaz BC, Braga-Campos FC, Badaró MIM (2019). O Programa de Volta para Casa na vida cotidiana dos seus beneficiários. Saude Soc.

[14] Bolonheis-Ramos RCM, Boarini ML (2015). Comunidades terapêuticas: “novas” perspectivas e propostas higienistas. Hist Cienc Saude Manguinhos.

[15] Barreto KI de S, Filho GSG, Apolinário GS, Perrone PAK, Guirado LR, Laranjeira R (2021). Comunidade Terapêutica como parte da rede de atenção psicossocial: conformidade e monitoramento são possíveis?. Cad Defensoria Pública Estado de São Paulo.

[16] Bezerra e Silva ML, Dimenstein MDB (2014). Arquivos brasileiros de psicologia universidade Federal do Rio de Janeiro Brasil. Arq Bras Psicol.

[17] Campos RTO (2019). Saúde mental no Brasil: avanços, retrocessos e desafios. Cad Saude Publica.

[18] Macedo JP, de Abreu MM, Fontenele MG, Dimenstein M (2017). A regionalização da saúde mental e os novos desafios da reforma psiquiátrica brasileira. Saúde e Sociedade.

[19] Mota DCB, Silveira CM, Siu E, Gomide HP, Guerra LHA, Ronzani TM (2019). Estimating service needs for alcohol and other drug users according to a tiered framework: the case of the São Paulo, Brazil, metropolitan area. J Stud Alcohol Drugs Suppl.

[20] Instituto Brasileiro de Geografia e Estatística (IBGE) (2013). Pesquisa Nacional de Saúde.

[21] Coutinho ESF, França-Santos D, da Silva Magliano E, Bloch KV, Barufaldi LA, de Freitas Cunha C (2016). ERICA: patterns of alcohol consumption in Brazilian adolescents. Rev Saude Publica.

[22] Zucker RA, Donovan JE, Masten AS, Mattson ME, Moss HB (2008). Early developmental processes and the continuity of risk for underage drinking and problem drinking. Pediatrics.

[23] Bujalski M, Moskalewicz J, Stokwiszewski J (2021). Occupational position and alcohol use disorders in Poland. Int J Occup Med Environ Health.

[24] Kim JY, Asrani SK, Shah ND, Kim WR, Schneekloth TD (2012). Hospitalization for underage drinkers in the United States. J Adolescent Health.

[25] Erol A, Karpyak VM (2015). Sex and gender-related differences in alcohol use and its consequences: contemporary knowledge and future research considerations. Drug Alcohol Depend.

[26] Faller S, Peuker AC, Sordi A, Stolf A, Souza-Formigoni ML, Cruz MS (2014). Who seeks public treatment for substance abuse in Brazil? Results of a multicenter study involving four Brazilian state capitals. Trends Psychiatry Psychother.

[27] Bastos FIPM, Vasconcellos MTL, Boni RB, Reis NB, Coutinho CFDS (2017). III Levantamento Nacional Sobre o Uso de Drogas pela População Brasileira.

[28] McHugh RK, Votaw VR, Sugarman DE, Greenfield SF (2018). Sex and gender differences in substance use disorders. Clin Psychol Rev.

[29] O'connell H, Chin AV, Cunningham C, Lawlor B (2003). Alcohol use disorders in elderly people-redefining an age old problem in old age. BMJ.

[30] Witkiewitz K, Litten RZ, Leggio L (2019). Advances in the science and treatment of alcohol use disorder. Sci Adv.

[31] Curry SJ, Krist AH, Owens DK, Barry MJ, Caughey AB, Davidson KW (2018). Screening and behavioral counseling interventions to reduce unhealthy alcohol use in adolescents and adults: US Preventive Services Task Force Recommendation Statement. JAMA.

[32] Worley J (2021). Alcohol use disorder providing better care. J Psychosoc Nurs Ment Health Serv.

